# Repellent efficacy of 20 essential oils on *Aedes aegypti* mosquitoes and *Ixodes scapularis* ticks in contact-repellency assays

**DOI:** 10.1038/s41598-023-28820-9

**Published:** 2023-01-30

**Authors:** Hailey A. Luker, Keyla R. Salas, Delaram Esmaeili, F. Omar Holguin, Harley Bendzus-Mendoza, Immo A. Hansen

**Affiliations:** 1 Department of Biology, New Mexico State University, 1200 S. Horseshoe Dr., Las Cruces, NM 88003 USA; 2 Department of Plant and Environmental Sciences, New Mexico State University, Skeen Hall, Las Cruces, NM 88003 USA; 3 Department of Computer Science, New Mexico State University, 1290 Frenger Mall, Las Cruces, NM 88003 USA; 4 Institute for Applied Biosciences, New Mexico State University, 1200 S. Horseshoe Dr., Las Cruces, NM 88003 USA

**Keywords:** Animal behaviour, Entomology, Infectious diseases

## Abstract

Cases of mosquito- and tick-borne diseases are rising worldwide. Repellent products can protect individual users from being infected by such diseases. In a previous study, we identified five essential oils that display long-distance mosquito repellency using a Y-tube olfactometer assay. In the current study, the contact repellent efficacy of 20 active ingredients from the Environmental Protection Agency’s (EPA) Minimum Risk Pesticides list were tested using *Aedes aegypti* and *Ixodes scapularis*. We utilized an arm-in-cage assay to measure complete protection time from mosquito bites for these active ingredients. To measure tick repellency, we used an EPA-recommended procedure to measure the complete protection time from tick crossings. We found that of the 20 ingredients tested, 10% v/v lotion emulsions with clove oil or cinnamon oil provided the longest protection from both mosquito bites and tick crossings. We conclude that in a 10% v/v emulsion, specific active ingredients from the EPA Minimum Risk Pesticides list can provide complete protection from mosquito bites and tick crossings for longer than one hour.

## Introduction

Diseases transmitted by mosquitoes and ticks impact hundreds of millions of human lives annually^1–3^. Insect repellents, when applied correctly, are an effective preemptive tool to minimize and avoid the spread of mosquito- and tick-borne diseases^4,5^. Common commercially available repellents rely heavily on synthetically-derived active ingredients such as DEET, picaridin, IR3535, or PMD^5^. While these synthetically-derived repellents are effective and long-lasting, there has been increasing consumer interest in naturally-derived repellents^6–9^. In 1996, the Environmental Protection Agency (EPA) began publishing lists of active and inactive ingredients that are approved for use in ‘minimum risk pesticide products’^10^. In the United States, insect repellents are regulated and categorized together with pesticides by the EPA. These minimum-risk ingredients are registered under the Federal Insecticide, Fungicide, Rodenticide Act (FIFRA) section 25(b), and in this study, we refer to this list of ingredients as the ‘EPA 25(b) list’. Insect repellents and pesticides that consist of ingredients from the EPA 25(b) list are exempt from federal registration with the EPA. Registering a new insect repellent or pesticide with the EPA is a time-intensive and expensive process that is not feasible for many small or start-up businesses. Therefore, there is great appeal for these businesses to produce repellent or pesticide products using only active and inactive ingredients from the EPA 25(b) list^11^. Many of the ingredients from the EPA 25(b) list have not been systematically explored in the following assays and their complete protection time has not yet been determined.

In 2020, we conducted a study on the repellent efficacy of 21 active ingredients from the EPA 25(b) list, 19 of these ingredients were essential oils. We used a Y-tube olfactometer assay that measures mosquito attraction to a human hand in the presence or absence of a treatment. A significant reduction in mosquito attraction to a human hand in the presence of a treatment indicates the treatment provides long-distance mosquito repellency. For this study, ingredients were pipetted onto a cotton ball that was located between a researcher’s hand and the Y-tube apparatus. We measured mosquito attraction to the hand in the presence and absence of each treatment in 30 min intervals. We found that five of the 21 ingredients tested from the EPA 25(b) list significantly reduced mosquito attraction. Spearmint oil and garlic oil reduced mosquito attraction for 30 min, peppermint oil and lemongrass oil reduced attraction for 60 min, and cinnamon oil reduced attraction for 120 min^12^.

The Y-tube olfactometer assay measures long-distance repellency that occurs when an active ingredient interferes with mosquito olfaction and deters mosquito host-seeking behavior^13,14^. The olfactory system of mosquitoes consists of two organs, a pair of antennae and a pair of maxillary palps^15^. Both organs play a role in a mosquito’s ability to detect vaporized odorants when host-seeking and are the primary organs affected by insect repellents^16–18^. However, once a mosquito makes direct contact with a host, other sensory organs like the labella and tarsi are also utilized. Labella and tarsi have an array of sensory receptors that allow the mosquito to ‘taste’^19,20^. When a mosquito comes in contact with a host it can detect molecules present in the oily top layer of the host’s skin in addition to short, vaporized odor molecules^21,22^. Different active ingredients may present different modes of repellency that cannot be measured using the Y-Tube assay. Here we hypothesize, that a different subset of the previously tested active ingredients, from the EPA 25(b) list, will exhibit repellent properties when tested in a contact-repellency assay. To test this hypothesis, we performed arm-in-cage assays to measure the complete protection time (CPT) of each active ingredient in a 10% v/v lotion emulsion using the yellow fever mosquito, *Aedes aegypti*.

Like mosquitoes, ticks rely on blood from vertebrate hosts to complete their life cycle. Ticks also use similar cues like carbon dioxide emissions, body heat, and lactic acid to detect and locate their hosts^23,24^. Despite sharing these similarities in lifestyle, ticks and mosquitoes rely on different sensory organs and behaviors to detect and access a host. The tick olfactory system consists of a pair of front tarsi, that contain the Haller’s organ, and a pair of pedipalps^25,26^. The receptor proteins in these two structures differ from the mosquito sensory receptors found in antennae and maxillary palps. Chelicerata and Hexapoda are two subphyla of Arthropoda (ticks belonging to Chelicerata and mosquitoes to Hexapoda). The ancestors of Chelicerata and Hexapoda diverged about 300 million years ago from a common ancestor^27,28^. An important difference between these two subphyla is that Hexapoda have odorant, gustatory, and ionotropic receptors, whereas Chelicerata only have gustatory and ionotropic receptors^29–31^. Intriguingly, studies have found that many commercial insect repellents like DEET, picaridin, or IR3535, are effective repellents on both ticks and mosquitoes^32,33^. This raises the question if mosquitoes and ticks, two groups of organisms with different sensory organs and receptor proteins, will be repelled by the same active ingredients from the EPA 25(b) list. To answer this question, we conducted an EPA procedure using adult deer ticks, *Ixodes scapularis,* to determine the contact-repellent efficacy of the 20 different active ingredients.

It is important to note that the chemical composition of essential oils can vary depending on the manufacturer, production processes, the plant species, geographic location, and environmental conditions of the plant sources that are used for essential oil extracts^34–36^. For this study, we sourced all of our essential oils from Sigma-Aldrich® and specify the CAS number associated with each oil in Table 1.

**Table 1:** - Essential oils used in this study.

Chemical name	Millipore sigma #	CAS number	References
Castor oil	259853	8001-79-4	^37^
Cedarwood oil (Texas)	96090	8000-27-9	^38^
Cinnamon oil	W229202	8015-91-6	^39^
Citronella oil (Java)	W230812	8000-29-1	^40^
Clove oil	C8392	8000-34-8	^41^
Corn oil	C8267	8001-30-7	^42^
Cornmint oil	W521604	68917-18-0	^43^
Cottonseed oil	C7767	8001-29-4	^44^
Garlic oil	W250320	8000-78-0	^45^
Geraniol	163333	106-24-1	^46^
Geranium oil	W522104	8000-46-2	^47^
Lemongrass oil (East Indian)	W262404	8007-02-1	^48^
Linseed oil	430021	68553-15-1	^49^
Peppermint oil	77411	8006-90-4	^50^
2-Phenylethl propionate (2PEP)	W286702	122-70-3	^51^
Rosemary oil	W299200	8000-25-7	^52^
Sesame oil	S3547	8008-74-0	^53^
Soybean oil	S7381	8001-22-7	^54^
Spearmint oil	W303208	8008-79-5	^55^
Thyme oil	W306509	8007-46-3	^56^

## Materials and methods

### Mosquito culture

For all experiments conducted in this study, female *Aedes aegypti* from the UGAL (University of Georgia Laboratory) strain was used. This strain was donated from Alexander Raikhel’s laboratory at the University of California, Riverside. Batches of approximately 500 eggs were hatched in 33 × 51 × 5 cm pans containing three liters of deionized water. Cat pellets were fed to the larvae ad libitum. Pupae were sorted into 200 mL plastic cups and transferred to BugDorm-1 Insect Rearing Cages (30 × 30 × 30 cm, Bug dorm Company, Taichung, Taiwan). A 100 mL Erlenmeyer flask containing 20% sucrose solution with a cotton wick at the top was put in the cages and changed weekly. Cages were kept inside an insectary room that maintained a temperature of 27 °C and 80% humidity with a light/dark cycle of 14/10, respectively.

### Ticks

Adult females of *Ixodes scapularis* were obtained from the Oklahoma State University’s (OSU) Tick Rearing Facility. After arrival in the mail, ticks were transferred to sealed paper cups and were kept hydrated using a damp piece of paper. The ticks were stored in an insectary room that maintained a temperature of 27° C and 80% humidity with a light/dark cycle of 14/10, respectively.

### Formulations

All active ingredients used in this study were procured from Millipore Sigma (see Table 1). To produce a 10% v/v lotion emulsion, 0.5 mL of each essential oil was mixed with 4.5 mL of an organic lotion base (chemistrystore.com, Stephenson Organic Lotion Base, SKU: 82076) in a 14 mL falcon tube. The tubes were vortexed for sixty seconds at the highest speed. The unscented lotion base was used as a control in the contact-repellency assays.

### Ethics declarations

All experiments conducted in this study have been reviewed and approved by the New Mexico State University Institutional Review Board. Our current application—(22010) ‘Insect and Tick-Repellent Research’ expires 09/2023. All participants were given and signed an informed consent form. Vulnerable persons (i.e., minors, pregnant and nursing women, prisoners, immune-compromised individuals, and people with severe reactions to mosquito bites) were excluded from this study. Participants were advised to avoid alcohol, tobacco, and any scented products at least 12 h prior to this study.

### Mosquito arm-in-cage assay

**Figure 1:**
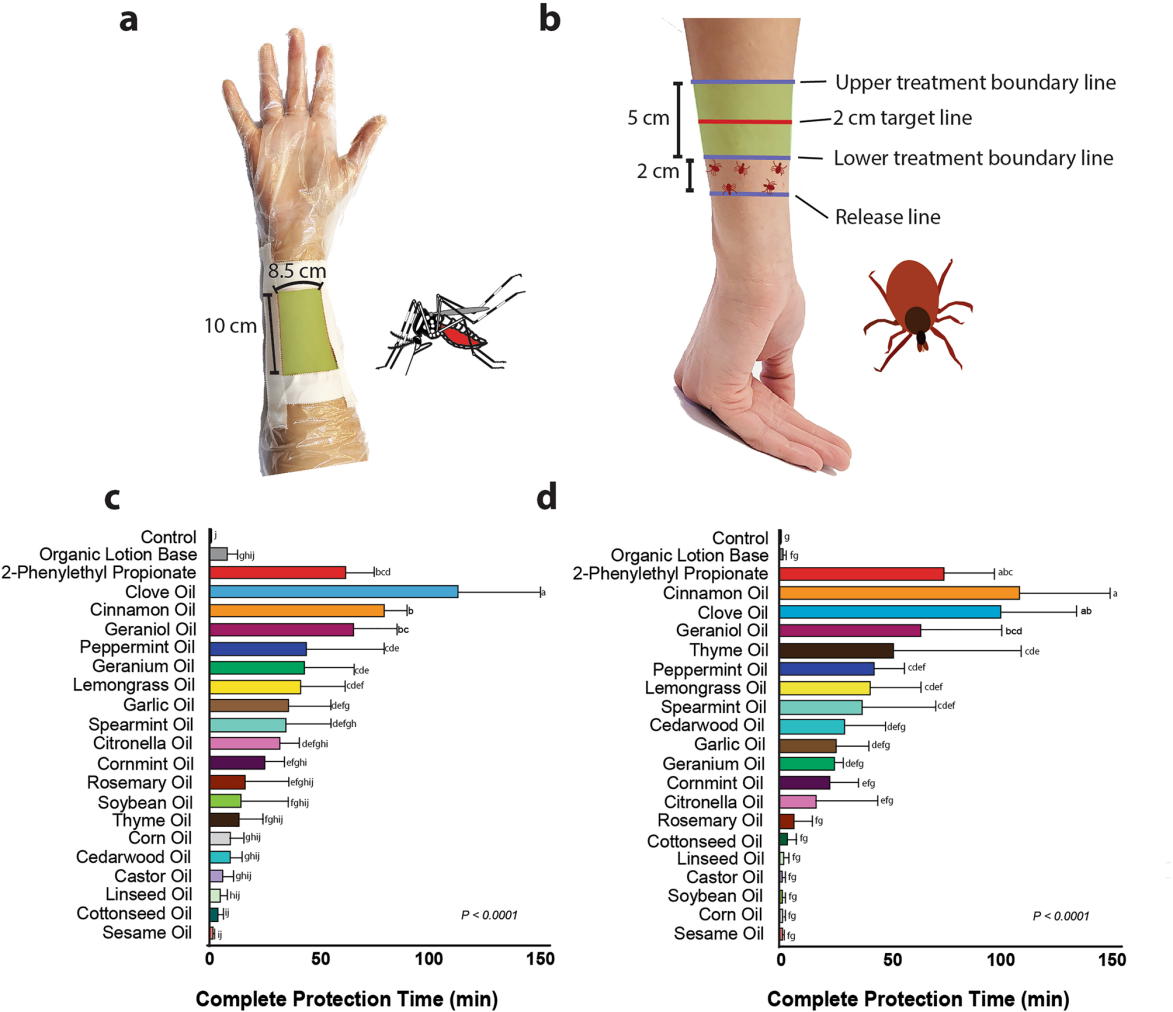
Contact-repellency assays: setup and results. (**a**) Experimental setup of the volunteer’s arm in the mosquito arm-in-cage assay. The green rectangle indicates the exposed skin surface that is either untreated or treated with a 10% v/v lotion emulsion. The rest of the volunteer’s hand and arm is covered with a plastic glove. (**b**) Experimental setup of the tick on-arm assay. Shown are four rings that are traced onto the volunteer’s forearm using a ballpoint pen. The shaded green portion indicates where the treatment was applied. The red-colored target line indicates the 3rd ring that is used to determine a tick crossing. (**c**) Complete protection times (CPTs) were measured with the mosquito arm-in-cage assay. An ANOVA test and Tukey’s multiple comparisons test was performed to determine statistical differences between different treatments. Experimental groups that do not share the same letter are significantly different from each other (*P* < 0.05). (**d**) CPTs measured with the tick on-arm assay. An ANOVA test and Tukey’s multiple pairwise-comparisons test were performed to determine statistical differences between different treatments. Experimental groups that do not share the same letter are significantly different from each other (*P* < 0.05).

This assay was conducted following the published guidelines of the U.S. Environmental Protection Agency for testing insect repellents that are applied to human skin^57^. 25 female mosquitoes that were one to two weeks old were transferred into a BugDorm cage (30 × 30 × 30 cm, Bug dorm Company, Taichung, Taiwan), modified with two plexiglass walls for observation. An elbow-length disposable polyethylene glove (Royal, Dayton, OH) was prepared by cutting out a rectangular-shaped area (8.5 × 10 cm) below the wrist on the inner forearm side (see Fig. 1a). The volunteer washed their forearm with an unscented soap, dried it with paper towels, and wiped their arm with an ethanol-soaked paper towel. Once the ethanol evaporated, the volunteer inserted their hand inside the prepared glove and the edges of the rectangular cutout were secured to the volunteer’s arm using cloth surgical tape. The volunteer then inserted their untreated forearm, as a control, into the mosquito infested BugDorm cage. A stopwatch was started. If one mosquito bite was recorded within 60 s, the experiment could proceed. After the volunteer received one bite during the control, they immediately shook off the mosquito and retracted their arm from the cage. We defined a mosquito bite as the insertion of a mosquito’s proboscis into a volunteer’s skin while probing for blood. 170 µl of a 10% v/v lotion emulsion was pipetted on the exposed area of the volunteer’s forearm and was spread thoroughly with the pipette tip. The end of each pipette tip was trimmed with scissors to reverse pipet the lotion emulsions. Once the treatment was applied to the volunteer a timer was started. The volunteer inserted their treated forearm into the same mosquito-infested cage and kept their arm in for 15 min or until the treatment failed. If after 15 min the treatment still provided protection, the volunteer retracted their arm from the cage and waited until the timer reached 30 min and began testing for 5 min intervals every 30 min until the treatment failed. This assay measures the complete protection time (CPT) of a treatment. The complete protection time was defined as the time from the application of a treatment up to when the volunteer received a 1st bite. The 1st bite must be confirmed by a confirmation bite (2nd bite) within 30 min. If 30 min passed without a confirmation bite, the time of the 1st bite was dismissed, and the volunteer continued testing. If a volunteer received a 1st bite at any point of the testing, they were asked to leave their arm in the cage for up to 30 min or until a confirmation bite was recorded.

### EPA procedure tick on-arm crawling assay

This assay was conducted according to the published guidelines of the U.S. Environmental Protection Agency for testing insect repellents that are applied to human skin^57^. Five female adult ticks were placed into a small plastic portion container with a lid prior to testing. The volunteer’s forearm was washed with an unscented soap and dried with a paper towel. A ballpoint pen was used to mark rings around the volunteer’s forearm. Four rings were drawn on the volunteer’s forearm (see Fig. 1b). The first ring was located above the wrist and is referred to as the release line. The second ring (lower treatment boundary line) was located 2 cm above the first ring. A third ring (2 cm target line) was drawn in the treatment area 2 cm away from the second line. This ring indicated the crossing point a tick must pass to be considered a tick crossing. The fourth ring (upper treatment boundary line) was located 5 cm above the second ring and indicated the ending boundary line for the treatment. Once the volunteer’s arm had all four rings drawn, a control test was performed. For the control, the volunteer placed their hand, palm-side up, on a white plastic pan, and five ticks were placed at the release line (1st ring). If one of the five ticks crossed the 2 cm target line within 60 s, the testing proceeded. If the control succeeded, the ticks were removed from the volunteer’s arm and the volunteer’s arm was treated with 250 µl of a 10% v/v lotion emulsion. The emulsion was spread on the volunteer’s arm between the second and fourth ring with a gloved finger. Once the treatment was applied, a timer was started, and the volunteer began testing. The treatments were tested for 5-min intervals every 30 min until a tick crossed the 2 cm target line. This assay was used to measure the complete protection time (CPT) of a treatment using tick crossings as the failure event. If a tick crossed 2 cm into the treatment zone (passed the 3rd ring), it is considered the first failure event. If a 2nd crossing did not occur within 30 min of the 1st crossing, we discarded the time point of the 1st crossing and continued testing.

### Statistical analysis

Complete protection times for the mosquito arm-in-cage assay and tick on-arm assay were compared between each treatment and control group using a one-way analysis of variance (ANOVA) and Tukey’s multiple pairwise-comparisons test. The significant differences between each group were determined using the p-value for each pairwise comparison. If the p-value was less than 0.05, the groups were significantly different. All tests were conducted in R statistical software (R Foundation for Statistical Computing, Vienna, Austria). The ANOVA and Tukey’s multiple pairwise-comparisons test were generated by using base R functions aov and TukeyHSD. Summary statistics on the results for each assay were generated by using the group_by and summary functions from the dplyr package. The compact letter display (to help report the results of each pairwise comparison) was generated by using the multcompLetters4 function within the multcompView package.

### Gas chromatography/mass spectrometry (GC/MS)

Each essential oil was diluted independently into a 10% solution in acetone. A sample containing equal volumes of each diluted essential oil was also created as an alignment file for the analysis. Diluted oils were analyzed by GC/MS as previously described by (Adams 2007) with the following adaptations a Varian model 3400 GC with an RTX-5 column (30 m × 0.25 mm fused silica capillary, 0.25 µm film thickness), coupled to a Saturn 2000 ion trap mass spectrometer (EI, 70 eV). Helium carrier gas flowed at 1 mL/min, and injector and transfer line temperatures were 270 and 180 °C, respectively. The initial column temperature was 60 °C, with a linear gradient of 3 °C/min.

Deconvolution and spectra processing was performed using Mass Spectrometry-Data independent Analysis software (MS-DIAL)^58^. Varian-generated MS files were converted into net CDF format using OpenChrom^59^ for rapid data retrieval in MS-DIAL. Automatic peak detection was performed on peaks with a minimum peak area of 500. For identification and alignment, we calculated Kovats retention index based on a C7-C40 saturated alkane mix Sigma Cat#49452-U and performed spectral matching with a cut-off of 70%, and calculated retention index tolerance of 50 to the Essential Oils GC/MS library^60^. Volatiles specific from garlic oil not present in the Adams mass spectral library were identified by retention index and the presence of a diagnostic ion as described in Satyal et al. ^61^. Peak alignment was performed using a reference sample that contained equal amounts of each oil.

## Results

### Effects of essential oil application on volunteers’ skin

Given the volatile nature and potential irritation hazard of essential oils^62^, we produced emulsions with an unscented lotion base for our experiments. Throughout this study, we carefully monitored the effects of each 10% v/v lotion formulation that was applied on to volunteers’ skin. We did not observe allergic or otherwise adverse skin reactions to any of the 20 different lotion emulsions with any of our volunteers. Volunteers reported that treatments containing spearmint oil or peppermint oil caused a cooling sensation on their skin.

### Repellent efficacy of different essential oils on* Aedes aegypti*

Figure 1c and Supplemental Table S1 show the complete protection times (CPTs) of different lotion formulations measured in the arm-in-cage assay. The lotion control protected from mosquito bites for approximately two minutes. The repellent efficacy of each 10% lotion formulation varied widely with CPTs ranging from 112 min to less than one minute. The 10% lotion formulations that provided the longest protection from mosquito bites were clove oil, cinnamon oil, 2PEP, and geraniol with CPTs over one hour. 10% lotion formulations with peppermint, geranium, lemongrass, garlic, spearmint, or citronella oil provided CPTs for longer than 30 min, while the rest of the lotion formulations did not provide significantly different CPTs compared to the untreated or unscented lotion controls. Our positive control, 10% DEET in unscented lotion, provided protection for up to six hours, at which time we stopped the experiment.

### Repellent efficacy of different essential oils on *Ixodes scapularis*

Figure 1d and Supplemental Table S2 show the complete protection times (CPTs) measured using the EPA procedure (tick on-arm assay) for different formulations. The organic lotion base provided no protection from tick crossings. The 10% lotion formulations that provided the longest protection from tick crossings were cinnamon oil, clove oil, 2PEP, and geraniol with CPTs longer than one hour. The formulation with 10% thyme oil displayed a CPT of 55 min, while the rest of the formulations tested did not provide CPTs significantly different from the untreated and unscented lotion controls. Our positive control, 10% DEET in unscented lotion, provided protection for up to six hours, at which time we stopped the experiment.

### GC/MS analysis of 20 essential oils

**Figure 2:**
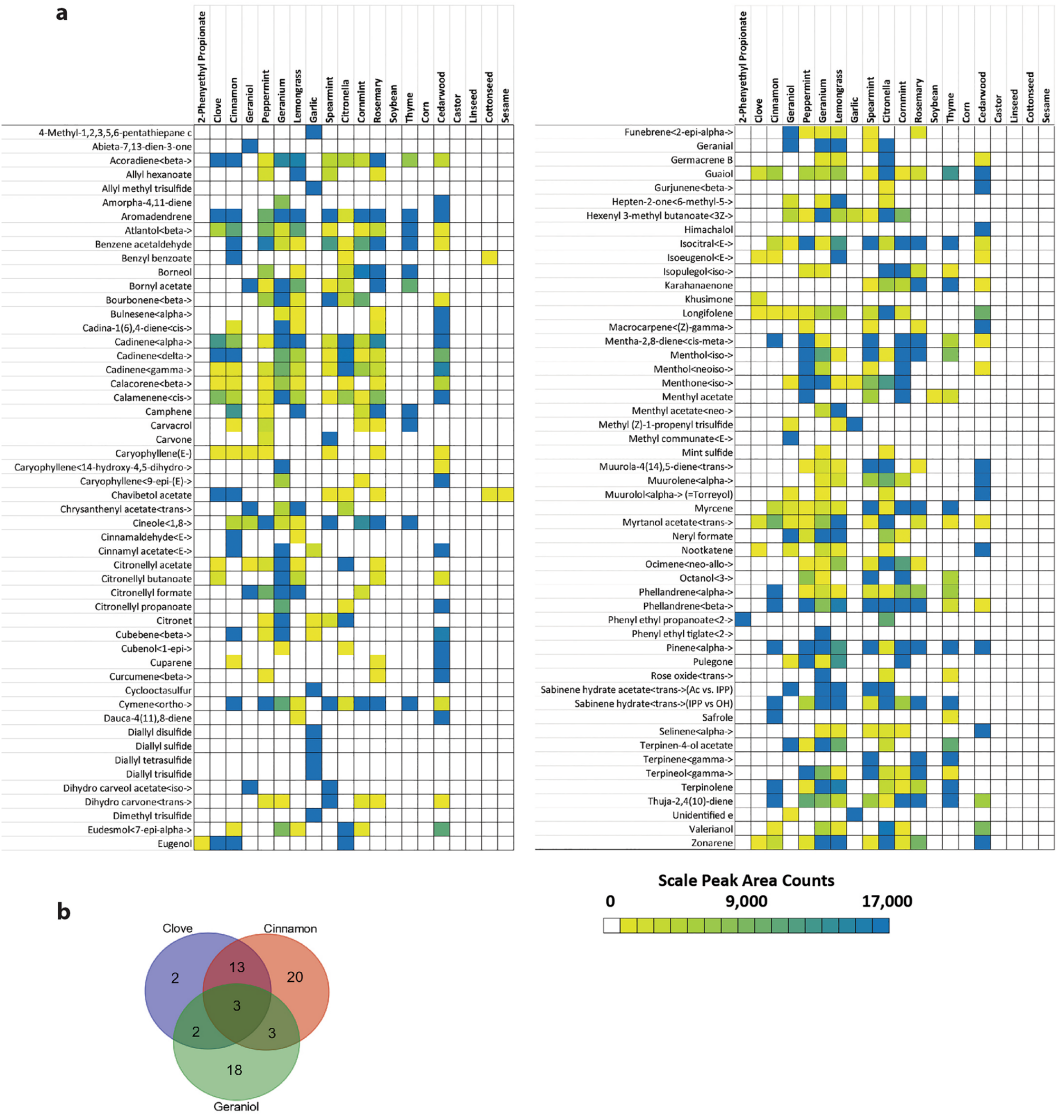
Terpene composition of various essential oils—Shown are the results of GC/MS analysis. (**a**) Heatmap of identified compounds in each essential oil. Peak areas for each compound are displayed based on the peak area of all identified components across each essential oil. Cells with a low or non-detectable component are represented in yellow and white, while deep blue represents components with relatively large peaks across all components and sample types. (**b**) Venn diagram of three top-performing essential oils. Amongst the three top-performing essential oils only 3 terpenes were common. Each essential oil uniquely contained terpene profiles whereas Cinnamon oil had 20 terpenes uniquely found followed by Geraniol with 18 and Clove with only 2 unique terpenes.

One hundred and five peaks were putatively identified, across all the essential oil samples. The top three best-performing emulsions for repelling *Aedes aegypti* mosquitoes and *Ixodes scapularis* ticks were emulsions with clove oil, cinnamon oil, and geraniol. These three essential oils contained 20, 39, and 26 identifiable terpenes. Of which only the following three terpenes were common to all three oils; Caryophhyllene (E-), Longifolene, and Myrtanol Acetate Fig. 2a. Clove oil contained two unique terpenes, Khusimone and Citronellyl butanoate, that differed from cinnamon oil and geraniol. While cinnamon oil and geraniol oil contained 20 and 18 unique compounds, respectively. The terpenes identified in each essential oil and their respective abundance is illustrated in Fig. 2b. See Supplemental Table S3 for a compound list with reported and expected retention index and spectrum similarity for this analysis.

## Discussion

Global warming is impacting the distribution ranges of many arthropod disease vectors, as a result increasing the number of people at risk of contracting vector-borne diseases^63,64^. Arthropod repellents can be an effective way to deter mosquitoes, ticks, and other blood-sucking arthropods from biting humans and transmitting pathogens^5^. However, the efficacy and duration of different repellent products can vary dramatically^65^. The type and concentration of active ingredient(s) in a repellent product is critical to the product’s efficacy at deterring mosquito and tick bites. While many of the active ingredients used in our study are known as essential oils and are widely used for aromatherapy and perfumery^66^, some of them are known to cause allergic contact dermatitis in sensitive persons^67^. Therefore, the US EPA recommends using low concentrations of these ingredients in products^10,67–69^.

There are many different experimental protocols to test the efficacy of mosquito and tick repellents. Each of these protocols measure different events to determine mosquito or tick repellency or ‘reduction in attraction’ of a product. The suitable protocol to use to test the repellent efficacy of a specific product depends on many variables including available resources, requirements for commercializing a product, the active ingredients, or the target arthropod. Some standard and commonly used protocols for repellency testing have been published by the US EPA, WHO, and various independent researchers. For mosquito contact repellency testing, the US EPA recommends testing pesticide products through field testing or an arm-in-cage assay both of which require a live volunteer and Institutional Review Board (IRB) approval^70^. While for testing spatial mosquito repellents, the WHO recommends a variety of assays that observe mosquito response to chemical stimulus through recording mosquito location within an assay^71,72^. To test tick repellents various assays have been developed some using live hosts or an artificial attractant^73^. Others, like the Tick Carousel Assay we previously developed to measure repellent efficacy by measuring tick engagements to a cloth, use the researcher’s carbon dioxide emissions and heat to indirectly attract ticks^74^. The US EPA recommends using a contact assay, that measures the location of ticks on a volunteer’s arm to calculate CPT^57^.

**Figure 3:**
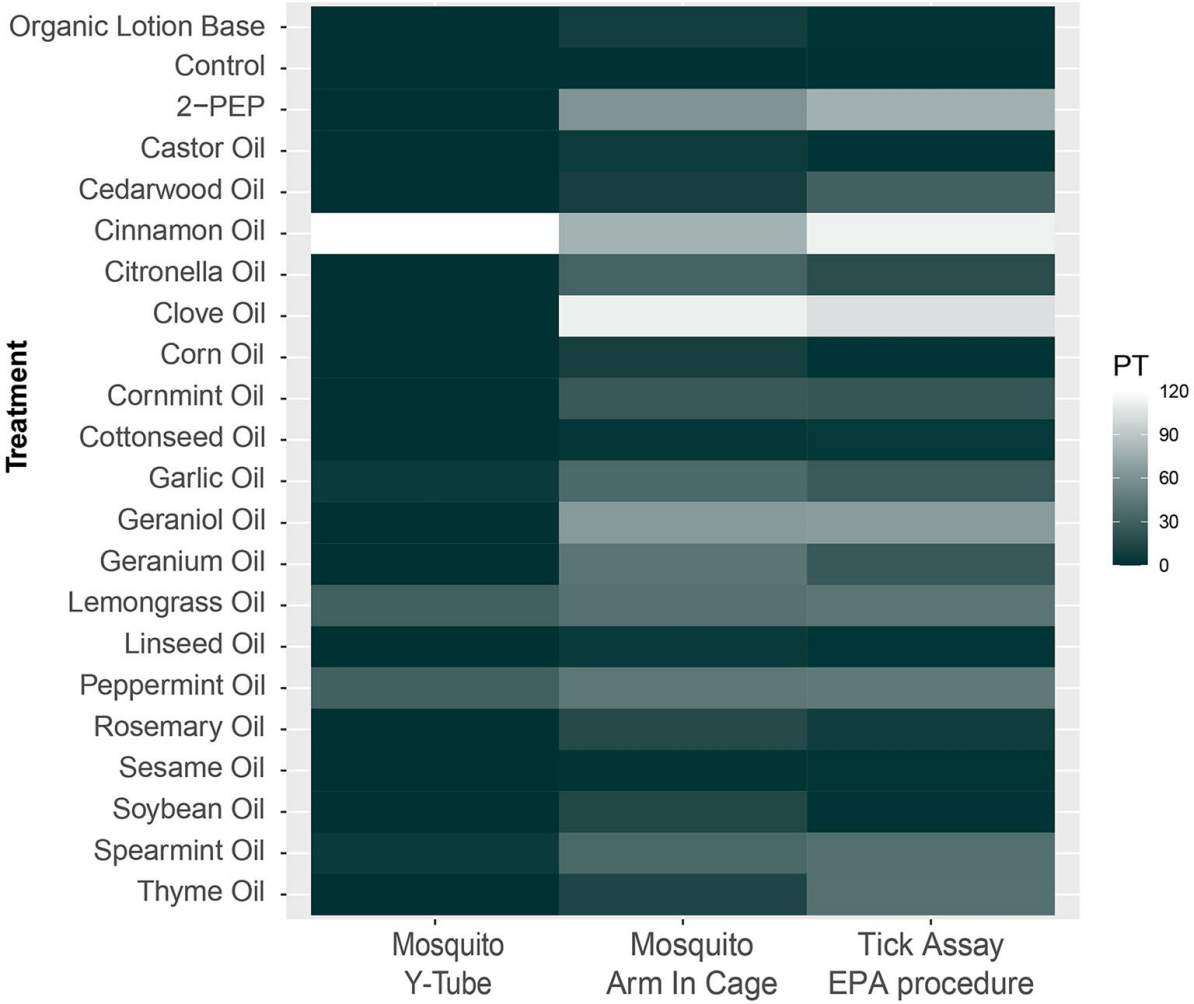
Protection times (PT) heat map. The protection time measured for specific active ingredients in three different tests is shown in minutes. The left column shows the results obtained from the long-distance mosquito repellency assay; the middle column shows the results of the mosquito contact repellency assay; the right column shows the results of the tick contact repellency assay. The colors represent the PT in minutes, darker color indicates a shorter or no PT while lighter color indicates longer PT.

In this study, we performed two of the above-mentioned protocols. We utilized the EPA’s “Specific guidance for laboratory studies of mosquito or biting fly repellency” (referred to as mosquito arm-in-cage protocol) and “Specific guidance for laboratory studies of tick and chigger repellency” (referred to as tick on-arm protocol) to test repellent efficacy of treatments on mosquitoes and ticks. In Fig. 3, we show a heat map that contains data from our previously conducted Y-tube olfactometer assay^75^ and our current results with mosquito and tick contact repellency assays. Of the treatments tested, only cinnamon oil displayed repellency in all three assays. Data collected from the Y-tube assay was determined not to be a useful predictor for how a treatment would perform in one of the contact repellency assays. For example, clove oil did not exhibit long-distance repellency in the Y-tube assay but significantly repelled both ticks and mosquitoes for more than 60 min in the contact assays. When comparing the results of both contact repellency assays, conducted on mosquitoes or ticks, we noticed that specific active ingredients displayed similar repellent efficacies on both organisms. Treatments with cinnamon oil, clove oil, and geraniol emulsions resulted in relatively long CPTs that were comparable in both mosquitoes and ticks. Since olfactory receptors are absent in tick genomes, gustatory and ionotropic receptors are probable candidates for this shared sensory response. However, an alternative explanation for this phenomenon is that these specific essential oils interact with multiple different olfactory receptor systems, including odorant receptors in mosquitoes. Further research is necessary to identify which receptor systems respond to these treatments in both mosquitoes and ticks.

The scientific literature on the repellent efficacy of essential oils on *Aedes aegypti* is often inconsistent. These deviations often appear to directly correlate with the concentration of the essential oil tested, and the type of solvent used to dilute the essential oil. Also, the chemical consistency of essential oils can be variable^34^. Factors, such as the location, environmental conditions, and specific plant species used for the extraction of essential oils may account for some of the inconsistencies found in the literature. The repellent efficacy of citronella oil is a useful example of how these two variables can impact the protection time of essential oils. Citronella oil applied to human skin resulted in reported protection times from mosquito bites that range from 9 to 120 min^76,77^. In three different studies that used an arm-in-cage assay to measure protection time, a 10% citronella oil emulsion in lotion, a 10%, 50%, and 100% citronella mixture with alcohol, and a 20% citronella oil in a complex solvent mixture were tested. In our study we found that 10% v/v citronella oil emulsions in a lotion base protected for 30 min. Trongtokit and collaborators found that 10% citronella oil in an alcohol solution provided no protection from mosquito bites, but 50% and 100% citronella oil solutions provided 50 min and 120 min of protection, respectively^78^. In a study conducted by Amer and Mehlhorn, a 20% citronella oil solution in a complex solvent mixture (20% Genapol, 10% Polyethylene Glycol, 20% ethanol, and 50% water) provided protection from mosquito bites for 120 minutes^77^. In our current study, we report that 10% emulsions in lotion with the essential oils Thyme, Cedarwood, Soybean, or Rosemary provide very short protection times of less than 20 min. Our findings would anticipate these four essential oils to be generally ineffective as a mosquito repellent, however this appears to not necessarily be the case. Amer and Mehlhorn also tested these four essential oils in a 20% dilution within their complex solvent and found significant protection times from *Aedes aegypti* bites for longer than 2 hours, with the rosemary solution providing protection for 5 hours^77^.

Based on our analyses, we hypothesize that the solvent used for an essential oil repellent product greatly determines an essential oil’s property to repel mosquitoes and protect from mosquito bites. Also, we propose that the source of an essential oil used in a repellent product may directly impact the repellent properties of the product. Further analysis is needed to test these hypotheses.

The terpene composition of the essential oils that we analyzed using GC/MS was intriguingly complex (see Fig. 2). Individual essential oils contained dozens of different terpenes. Terpenes are unsaturated hydrocarbons, that are volatile and often serve as odorants^79^. We found that essential oils that contain few terpenes, such as soybean oil, castor oil, linseed oil, cottonseed oil, and sesame oil, don’t repel mosquitoes or ticks. The best-performing repellent essential oils, cinnamon oil, and clove oil, have high similarity in their terpene composition. Almost all types of terpenes found in clove oil are also found in cinnamon oil. The third-best performing repellent essential oil—geraniol—shows little overlap in its terpene composition, with clove oil and cinnamon oil.

In summary, our findings show that several of the essential oils and other ingredients from the EPA’s 25(b) list are mosquito and tick repellents. Protection times of longer than 100 min were recorded for both mosquitoes and ticks, after the application of 10% lotion emulsions containing either cinnamon oil or clove oil. Future projects will address the identification and assessment of active ingredients within the repellent essential oils found in this study and the potential of synergistic effects when mixing these compounds. Our goal is to find a lotion emulsion with no more than 10% of active ingredients that can repel ticks and mosquitos for up to or longer than three hours. Lastly, we plan to characterize tick gustatory and ionotropic receptors through electrophysiology to begin to describe a mode-of-action for repellent components within the most repellent essential oils.

## Supplementary Information

Supplementary Information.

## Data Availability

All data generated or analyzed during this study are included in this published article (and its Supplemental Information files).
