# Supplementary material for: Repellent efficacy of 20 essential oils on Aedes aegypti mosquitoes and Ixodes scapularis ticks in contact-repellency assays
Source: Sci Rep. 2023 Jan 30;13:1705. doi: 10.1038/s41598-023-28820-9 (PMC9886999; doi:10.1038/s41598-023-28820-9)
Supplement: Supplementary file 1 — Supplementary Information. [file 41598_2023_28820_MOESM1_ESM.pdf]

**Supplemental Information** for "Repellent efficacy of 20 essential oils on *Aedes aegypti* mosquitoes and *Ixodes scapularis* ticks in contact-repellency assays" by Hailey A. Luker, Keyla R. Salas, Delaram Esmaeili, F. Omar Holguin, Harley Bendzus-Mendoza, & Immo A. Hansen

| | | | | | | | | | | | | | | | |
|---|---|---|---|---|---|---|---|---|---|---|---|---|---|---|---|
| **Necgative Control - Organic Lotion Base** | | | | **Positive Control - DEET** | | | | | | | | | | | |
| | | Treatment | | | | Treatment | | | | | | | | | |
| Trial | Control | 1st Bite | 2nd Bite | Trial | Control | 1st Bite | 2nd Bite | | | | | | | | |
| 1 | 0.3 | 9.13 | 10.4 | 1 | 0.22 | 6+ | 6+ | | | | | | | | |
| 2 | 0.97 | 3.07 | 9.44 | 2 | 0.42 | 6+ | 6+ | | | | | | | | |
| 3 | 0.92 | 5.07 | 5.88 | 3 | 0.55 | 6+ | 6+ | | | | | | | | |
| 4 | 0.62 | 13.55 | 15.08 | 4 | 0.15 | 6+ | 6+ | | | | | | | | |
| Average | 0.70 | 7.71 | 10.20 | Average | 0.34 | | | | | | | | | | |
| **CPT of Organic Lotion Base** | | | **7.71** | **CPT of DEET** | | | **6+** | | | | | | | | |
| **10% Castor Oil Emulsion** | | | | **10% Cedarwood Oil Emulsion** | | | | **10% Citronella Oil Emulsion** | | | | **10% Cinnamon Oil Emulsion** | | | |
| | | Treatment | | | | Treatment | | | | Treatment | | | | Treatment | |
| Trial | Control | 1st Bite | 2nd Bite | Trial | Control | 1st Bite | 2nd Bite | Trial | Control | 1st Bite | 2nd Bite | Trial | Control | 1st Bite | 2nd Bite |
| 1 | 0.72 | 2.55 | 4.82 | 1 | 0.32 | 3.67 | 6.45 | 1 | 0.08 | 26.4 | 33.28 | 1 | 0.47 | 87.48 | 91.5 |
| 2 | 0.58 | 4.17 | 4.35 | 2 | 0.55 | 16.47 | 17.42 | 2 | 0.38 | 38.22 | 41.22 | 2 | 0.32 | 77.25 | 84.5 |
| 3 | 0.6 | 2.97 | 10.22 | 3 | 0.05 | 8.47 | 13.44 | 3 | 0.15 | 39.22 | 39.43 | 3 | 0.57 | 64.23 | 66.67 |
| 4 | 0.48 | 12.95 | 20.7 | 4 | 0.05 | 7.5 | 15.28 | 4 | 0.12 | 21.57 | 23.18 | 4 | 0.73 | 84.13 | 87.12 |
| Average | 0.60 | 5.66 | 10.02 | Average | 0.24 | 9.03 | 13.15 | Average | 0.18 | 31.35 | 34.28 | Average | 0.52 | 78.27 | 82.45 |
| **CPT of 10% Castor Oil** | | | **5.66** | **CPT of 10% Cedarwood Oil** | | | **9.03** | **CPT of Citronella Oil** | | | **31.35** | **CPT of Cinnamon Oil** | | | **78.27** |
| **10% Clove Oil Emulsion** | | | | **10% Corn Oil Emulsion** | | | | **10% Cornmint Oil Emulsion** | | | | **10% Cottonsed Oil Emulsion** | | | |
| | | Treatment | | | | Treatment | | | | Treatment | | | | Treatment | |
| Trial | Control | 1st Bite | 2nd Bite | Trial | Control | 1st Bite | 2nd Bite | Trial | Control | 1st Bite | 2nd Bite | Trial | Control | 1st Bite | 2nd Bite |
| 1 | 0.32 | 158.92 | 160.82 | 1 | 0.48 | 3.4 | 3.9 | 1 | 0.38 | 28.87 | 31.63 | 1 | 0.3 | 2.43 | 4.63 |
| 2 | 0.2 | 104.22 | 104.52 | 2 | 0.15 | 11.1 | 12.57 | 2 | 0.37 | 25.75 | 37.67 | 2 | 0.18 | 4.25 | 7.2 |
| 3 | 0.45 | 120.12 | 121.18 | 3 | 0.85 | 16.78 | 17.17 | 3 | 0.33 | 11.88 | 14.85 | 3 | 0.07 | 6.6 | 9.77 |
| 4 | 0.57 | 56 | 58.33 | 4 | 0.88 | 5.05 | 6 | 4 | 0.42 | 31.83 | 37.83 | 4 | 0.52 | 0.83 | 5.63 |
| 5 | 0.4 | 117.98 | 125.52 | Average | 0.59 | 9.08 | 9.91 | Average | 0.38 | 24.58 | 30.50 | Average | 0.27 | 3.53 | 6.81 |
| Average | 0.39 | 111.45 | 114.07 | **CPT of Corn Oil** | | | **9.08** | **CPT of Cornmint Oil** | | | **24.58** | **CPT of Cottonseed Oil** | | | **3.53** |
| **CPT of Clove Oil** | | | **111.45** | | | | | | | | | | | | |
| **10% Garlic Oil Emulsion** | | | | **10% Geraniol Oil Emulsion** | | | | **10% Geranium Oil Emulsion** | | | | **10% Lemongrass Oil Emulsion** | | | |
| | | Treatment | | | | Treatment | | | | Treatment | | | | Treatment | |
| Trial | Control | 1st Bite | 2nd Bite | Trial | Control | 1st Bite | 2nd Bite | Trial | Control | 1st Bite | 2nd Bite | Trial | Control | 1st Bite | 2nd Bite |
| 1 | 0.23 | 9.85 | 12.52 | 1 | 0.08 | 38.25 | 38.53 | 1 | 0.83 | 33.63 | 34.73 | 1 | 0.13 | 38.17 | 41.23 |
| 2 | 0.9 | 63.6 | 67.72 | 2 | 0.27 | 63.5 | 65.82 | 2 | 0.32 | 35.15 | 35.33 | 2 | 0.47 | 34.78 | 36.63 |
| 3 | 0.13 | 32.5 | 33.78 | 3 | 0.78 | 93.32 | 94.32 | 3 | 0.2 | 12.2 | 20.87 | 3 | 0.6 | 52.4 | 53.67 |
| 4 | 0.63 | 35.12 | 43.5 | 4 | 0.27 | 60.73 | 67.8 | 4 | 0.73 | 68.38 | 68.32 | 4 | 0.4 | 12.35 | 15.08 |
| 5 | 0.88 | 35 | 39.17 | 5 | 0.25 | 66.5 | 68.67 | 5 | 0.2 | 62.57 | 68.08 | 5 | 0.38 | 65.62 | 74.28 |
| Average | 0.55 | 35.21 | 39.34 | Average | 0.33 | 64.46 | 67.03 | Average | 0.46 | 42.39 | 45.47 | Average | 0.40 | 40.66 | 44.18 |
| **CPT of Garlic Oil** | | | **35.21** | **CPT of Geranoil Oil** | | | **64.46** | **CPT of Geranium Oil** | | | **42.39** | **CPT of Lemongrass Oil** | | | **40.66** |
| **10% Linseed Oil Emulsion** | | | | **10% Peppermint Oil Emulsion** | | | | **10% 2-Phenylethyl Propionate Oil Emulsion** | | | | **10% Rosemary Oil Emulsion** | | | |
| | | Treatment | | | | Treatment | | | | Treatment | | | | Treatment | |
| Trial | Control | 1st Bite | 2nd Bite | Trial | Control | 1st Bite | 2nd Bite | Trial | Control | 1st Bite | 2nd Bite | Trial | Control | 1st Bite | 2nd Bite |
| 1 | 0.25 | 4.05 | 4.57 | 1 | 0.23 | 11.18 | 11.92 | 1 | 0.67 | 60.13 | 62.7 | 1 | 0.77 | 8.32 | 8.7 |
| 2 | 0.42 | 1.95 | 2.07 | 2 | 0.42 | 36.85 | 38.83 | 2 | 0.45 | 61.4 | 61.83 | 2 | 0.35 | 7.75 | 10.98 |
| 3 | 0.4 | 5.3 | 7.13 | 3 | 0.53 | 15.62 | 25.27 | 3 | 0.7 | 45.45 | 47.5 | 3 | 0.53 | 50.55 | 53.75 |
| 4 | 0.28 | 8.58 | 9.53 | 4 | 0.97 | 97.32 | 98.18 | 4 | 0.98 | 76.85 | 77.73 | 4 | 0.63 | 9.95 | 10.43 |
| Average | 0.34 | 4.97 | 5.83 | 5 | 0.42 | 55.33 | 55.63 | Average | 0.70 | 60.96 | 62.44 | 5 | 0.5 | 2.18 | 4.4 |
| **CPT of Linseed Oil** | | | **4.97** | Average | 0.51 | 43.26 | 45.97 | **CPT of 2-Phenylethyl Propionate Oil** | | | **60.96** | Average | 0.56 | 15.75 | 17.65 |
| | | | | **CPT of Peppermint Oil** | | | **43.26** | | | | | **CPT of Rosemary Oil** | | | **15.75** |
| **10% Sesame Oil Emulsion** | | | | **10% Soybean Oil Emulsion** | | | | **10% Spearmint Oil Emulsion** | | | | **10% Thyme Oil Emulsion** | | | |
| | | Treatment | | | | Treatment | | | | Treatment | | | | Treatment | |
| Trial | Control | 1st Bite | 2nd Bite | Trial | Control | 1st Bite | 2nd Bite | Trial | Control | 1st Bite | 2nd Bite | Trial | Control | 1st Bite | 2nd Bite |
| 1 | 0.2 | 1.75 | 3.28 | 1 | 0.17 | 1.58 | 1.83 | 1 | 0.35 | 34.37 | 62.22 | 1 | 0.25 | 12.38 | 23.83 |
| 2 | 0.53 | 1.67 | 2.3 | 2 | 0.47 | 51.37 | 51.63 | 2 | 0.25 | 31.32 | 32.58 | 2 | 0.23 | 30.45 | 31.97 |
| 3 | 0.98 | 1.37 | 10.48 | 3 | 0.52 | 2.43 | 3.13 | 3 | 0.08 | 8.43 | 11.33 | 3 | 0.8 | 13.63 | 37.3 |
| 4 | 0.83 | 2.13 | 3.02 | 4 | 0.73 | 3.72 | 9.97 | 4 | 0.22 | 30.93 | 32.6 | 4 | 0.17 | 2.5 | 15.75 |
| Average | 0.64 | 1.73 | 4.77 | 5 | 0.25 | 10.37 | 11.55 | 5 | 0.32 | 65.25 | 65.47 | 5 | 0.2 | 6.03 | 7.77 |
| **CPT of Sesame Oil** | | | **1.73** | Average | 0.43 | 13.89 | 15.62 | Average | 0.24 | 34.06 | 40.84 | Average | 0.33 | 13.00 | 23.32 |
| | | | | **CPT of Soybean Oil** | | | **13.89** | **CPT of Spearmint Oil** | | | **34.06** | **CPT of Thyme Oil** | | | **13.00** |

**Supplemental Table S1** – Raw data in minutes collected for the mosquito arm-in-cage assay

**Negative Control with Organic Lotion**

| Trial | 1st Crossing | 2nd Crossing |
|---|---|---|
| 1 | 0.63 | 1.1.67 |
| 2 | 0.90 | 1.83 |
| 3 | 1.98 | 3.65 |
| 4 | 1.82 | 2.92 |
| **Average** | **1.33** | **2.80** |
| CPT of Negat | 1.33 | |
| | 1min 20sec | |

**10% DEET Emulsion**

| Trial | Control 1st Crossing | Control 2nd Crossing | Treatment 1st Crossing | Treatment 2nd Crossing |
|---|---|---|---|---|
| 1 | 0.22 | 0.57 | | |
| 2 | 0.80 | 1.23 | 360.00 | 360.00 |
| 3 | 0.90 | 1.32 | 360.00 | 360.00 |
| 4 | 0.27 | 0.92 | 360.00 | 360.00 |
| Average | 0.55 | 1.01 | 360.00 | 360.00 |
| CPT of 10% DEET | | | 360.00 | |

**10% Castor Oil Emulsion**

| Trial | Control 1st Crossing | Control 2nd Crossing | Treatment 1st Crossing | Treatment 2nd Crossing |
|---|---|---|---|---|
| 1 | 0.87 | 2.58 | 3.30 | 3.47 |
| 2 | 0.73 | 6.59 | 1.08 | 4.72 |
| 3 | 0.63 | 1.67 | 2.67 | 7.00 |
| 4 | 0.90 | 1.83 | 2.28 | 3.47 |
| Average | 0.78 | 3.17 | 2.33 | 4.67 |
| CPT of 10% Castor Oil | | | 2.33 | |
| | | | 2min 20sec | |

**10% Citronella Oil Emulsion**

| Trial | Control 1st Crossing | Control 2nd Crossing | Treatment 1st Crossing | Treatment 2nd Crossing |
|---|---|---|---|---|
| 1 | 0.12 | 0.97 | 26.1 | 29.97 |
| 2 | 0.17 | 2.62 | 72.8 | 74.47 |
| 3 | 0.12 | 0.47 | 1.27 | 3.62 |
| 4 | 0.15 | 0.37 | 0.87 | 21.47 |
| 5 | 0.57 | 1 | 2.92 | 3.4 |
| 6 | 0.27 | 0.33 | 0.92 | 1.68 |
| Average | 0.233333333 | 0.96 | 17.48 | 22.435 |
| CPT of 10% Citonella Oil | | | 17.48 | |
| | | | 17min 29sec | |

**10% Garlic Oil Emulsion**

| Trial | Control 1st Crossing | Control 2nd Crossing | Treatment 1st Crossing | Treatment 2nd Crossing |
|---|---|---|---|---|
| 1 | 0.15 | 0.87 | 28.33 | 29.25 |
| 2 | 0.48 | 0.50 | 40.87 | 41.28 |
| 3 | 0.65 | 0.65 | 30.08 | 34.97 |
| 4 | 0.38 | 0.38 | 1.43 | 3.32 |
| 5 | 0.20 | 0.52 | 30.68 | 32.28 |
| Average | 0.37 | 0.58 | 26.28 | 28.22 |
| CPT of 10% Garlic Oil | | | 26.28 | |
| | | | 26min 17sec | |

**10% Linseed Oil Emulsion**

| Trial | Control 1st Crossing | Control 2nd Crossing | Treatment 1st Crossing | Treatment 2nd Crossing |
|---|---|---|---|---|
| 1 | 0.12 | 0.25 | 4.70 | 21.97 |
| 2 | 0.08 | 0.25 | 0.88 | 23.53 |
| 3 | 0.35 | 0.63 | 0.60 | 0.67 |
| 4 | 0.15 | 0.55 | 4.25 | 4.72 |
| Average | 0.18 | 0.42 | 2.61 | 12.72 |
| CPT of 10% Linseed Oil | | | 2.61 | |
| | | | 2min 37sec | |

**10% Cedarwood Oil Emulsion**

| Trial | Control 1st Crossing | Control 2nd Crossing | Treatment 1st Crossing | Treatment 2nd Crossing |
|---|---|---|---|---|
| 1 | 0.68 | 2.90 | 4.62 | 5.20 |
| 2 | 0.97 | 2.55 | 28.87 | 30.00 |
| 3 | 0.83 | 1.83 | 31.90 | 32.30 |
| 4 | 0.70 | 3.07 | 59.82 | 64.20 |
| 5 | 0.30 | 0.52 | 25.73 | 25.83 |
| Average | 0.70 | 2.17 | 30.19 | 31.51 |
| CPT of Cedarwood Oil | | | 30.19 | |
| | | | 30min 11sec | |

**10% Clove Oil Emulsion**

| Trial | Control 1st Crossing | Control 2nd Crossing | Treatment 1st Crossing | Treatment 2nd Crossing |
|---|---|---|---|---|
| 1 | 0.37 | 1.33 | 114.25 | 115.75 |
| 2 | 0.08 | 2.60 | 113.23 | 121.38 |
| 3 | 0.23 | 0.45 | 116.87 | 118.48 |
| 4 | 0.22 | 0.40 | 25.38 | 26.13 |
| 5 | 0.58 | 0.68 | 149.50 | 150.43 |
| Average | 0.30 | 1.09 | 103.85 | 106.43 |
| CPT of 10% Clove Oil | | | 103.85 | |
| | | | 1hour 43min 53sec | |

**10% Geranoil Oil Emulsion**

| Trial | Control 1st Crossing | Control 2nd Crossing | Treatment 1st Crossing | Treatment 2nd Crossing |
|---|---|---|---|---|
| 1 | 0.07 | 0.08 | 25.50 | 29.78 |
| 2 | 0.52 | 1.42 | 91.53 | 91.95 |
| 3 | 0.12 | 1.12 | 116.12 | 118.45 |
| 4 | 0.13 | 0.53 | 33.23 | 34.15 |
| 5 | 0.50 | 0.90 | 61.50 | 62.75 |
| Average | 0.27 | 0.81 | 65.58 | 67.42 |
| CPT of 10% Gereanoil Oil | | | 65.58 | |
| | | | 1hour 5min 35sec | |

**10% Peppermint Oil Emulsion**

| Trial | Control 1st Crossing | Control 2nd Crossing | Treatment 1st Crossing | Treatment 2nd Crossing |
|---|---|---|---|---|
| 1 | 0.58 | 0.87 | 55.15 | 55.18 |
| 2 | 0.20 | 0.43 | 58.88 | 59.49 |
| 3 | 0.75 | 0.80 | 30.27 | 30.30 |
| 4 | 0.32 | 0.45 | 34.02 | 0.93 |
| Average | 0.46 | 0.64 | 44.58 | 36.48 |
| CPT of 10% Peppermint Oil | | | 44.58 | |
| | | | 44min 35sec | |

**10% Cinnamon Oil Emulsion**

| Trial | Control 1st Crossing | Control 2nd Crossing | Treatment 1st Crossing | Treatment 2nd Crossing |
|---|---|---|---|---|
| 1 | 0.12 | 1.98 | 182.88 | 185.30 |
| 2 | 0.85 | 1.22 | 88.53 | 112.28 |
| 3 | 0.53 | 1.40 | 90.78 | 90.30 |
| 4 | 0.12 | 1.07 | 86.68 | 105.25 |
| Average | 0.41 | 1.42 | 112.22 | 123.28 |
| CPT of 10% Cinnamon Oil Emulsion | | | 112.22 | |
| | | | 1hour 52min 13sec | |

**10% Corn Oil Emulsion**

| Trial | Control 1st Crossing | Control 2nd Crossing | Treatment 1st Crossing | Treatment 2nd Crossing |
|---|---|---|---|---|
| 1 | 0.45 | 2.63 | 1.55 | 2.43 |
| 2 | 0.90 | 1.10 | 1.70 | 1.97 |
| 3 | 0.18 | 4.57 | 3.57 | 4.87 |
| 4 | 0.13 | 2.82 | 1.78 | 3.15 |
| Average | 0.42 | 2.78 | 2.15 | 3.11 |
| CPT of 10% Corn Oil | | | 2.15 | |
| | | | 2min 9sec | |

**10% Geranium Oil Emulsion**

| Trial | Control 1st Crossing | Control 2nd Crossing | Treatment 1st Crossing | Treatment 2nd Crossing |
|---|---|---|---|---|
| 1 | 0.05 | 0.15 | 20.65 | 28.90 |
| 2 | 0.57 | 0.88 | 30.75 | 55.57 |
| 3 | 0.32 | 0.48 | 23.97 | 29.10 |
| 4 | 0.12 | 0.37 | 28.17 | 53.42 |
| Average | 0.27 | 0.47 | 25.89 | 41.75 |
| CPT of 10% Geranium Oil | | | 25.89 | |
| | | | 25min 53sec | |

**10% 2-phenylethyl propionate Oil Emulsion**

| Trial | Control 1st Crossing | Control 2nd Crossing | Treatment 1st Crossing | Treatment 2nd Crossing |
|---|---|---|---|---|
| 1 | 0.12 | 0.42 | 85.20 | 85.65 |
| 2 | 0.42 | 0.48 | 57.10 | 57.40 |
| 3 | 0.20 | 0.53 | 61.25 | 61.67 |
| 4 | 0.12 | 0.42 | 107.41 | 108.52 |
| Average | 0.22 | 0.46 | 77.74 | 78.31 |
| CPT of 10% 2-phenylethl propionate Oil | | | 77.74 | |
| | | | 1 hour 17min 44sec | |

**10% Cornmint Oil Emulsion**

| Trial | Control 1st Crossing | Control 2nd Crossing | Treatment 1st Crossing | Treatment 2nd Crossing |
|---|---|---|---|---|
| 1 | 0.23 | 0.37 | 25.33 | 26.45 |
| 2 | 0.10 | 0.18 | 0.83 | 27.92 |
| 3 | 0.48 | 0.88 | 29.17 | 30.78 |
| 4 | 0.22 | 0.53 | 29.32 | 45.77 |
| 5 | 0.18 | 0.50 | 30.95 | 32.87 |
| Average | 0.24 | 0.49 | 23.12 | 32.76 |
| CPT of 10% Cornmint Oil | | | 23.12 | |
| | | | 23min 7 sec | |

**10% Cottonseed Oil Emulsion**

| Trial | Control 1st Crossing | Control 2nd Crossing | Treatment 1st Crossing | Treatment 2nd Crossing |
|---|---|---|---|---|
| 1 | 0.13 | 0.28 | 0.83 | 1.05 |
| 2 | 0.10 | 0.33 | 10.05 | 10.22 |
| 3 | 0.08 | 0.85 | 2.95 | 3.45 |
| 4 | 0.32 | 0.80 | 1.98 | 3.60 |
| Average | 0.16 | 0.57 | 3.95 | 4.58 |
| CPT of 10% Cottonseed Oil | | | 3.95 | |
| | | | 3min 57sec | |

**10% Lemongrass Oil Emulsion**

| Trial | Control 1st Crossing | Control 2nd Crossing | Treatment 1st Crossing | Treatment 2nd Crossing |
|---|---|---|---|---|
| 1 | 0.12 | 0.52 | 29.38 | 30.15 |
| 2 | 0.30 | 1.37 | 40.37 | 45.38 |
| 3 | 0.87 | 1.30 | 52.98 | 66.08 |
| 4 | 0.28 | 0.48 | 1.92 | 5.15 |
| 5 | 0.67 | 0.98 | 71.02 | 71.03 |
| 6 | 0.20 | 0.33 | 60.92 | 66.87 |
| Average | 0.41 | 0.83 | 42.77 | 47.44 |
| CPT of 10% Lemongrass Oil | | | 42.77 | |
| | | | 42min 46sec | |

**10% Rosemary Oil Emulsion**

| Trial | Control 1st Crossing | Control 2nd Crossing | Treatment 1st Crossing | Treatment 2nd Crossing |
|---|---|---|---|---|
| 1 | 0.18 | 0.42 | 3.30 | 30.72 |
| 2 | 0.32 | 0.88 | 1.98 | 18.97 |
| 3 | 0.55 | 1.05 | 19.13 | 23.65 |
| 4 | 0.72 | 1.95 | 3.17 | 4.68 |
| Average | 0.44 | 1.08 | 6.90 | 19.51 |
| CPT of 10% Rosemary Oil | | | 6.90 | |
| | | | 6min 54sec | |

**10% Sesame Oil Emulsion**

| Trial | Control 1st Crossing | Control 2nd Crossing | Treatment 1st Crossing | Treatment 2nd Crossing |
|---|---|---|---|---|
| 1 | 0.08 | 0.28 | 0.82 | 0.95 |
| 2 | 0.13 | 0.67 | 3.00 | 4.35 |
| 3 | 0.60 | 1.03 | 2.98 | 4.68 |
| 4 | 0.18 | 0.55 | 0.62 | 1.52 |
| Average | 0.25 | 0.63 | 1.86 | 2.88 |
| CPT of 10% Sesame Oil | | | 1.86 | |
| | | | 1min 52sec | |

**10% Soybean Oil Emulsion**

| Trial | Control 1st Crossing | Control 2nd Crossing | Treatment 1st Crossing | Treatment 2nd Crossing |
|---|---|---|---|---|
| 1 | 0.15 | 0.42 | 1.88 | 2.97 |
| 2 | 0.20 | 0.58 | 2.87 | 3.58 |
| 3 | 0.12 | 0.53 | 0.85 | 3.25 |
| 4 | 0.12 | 0.53 | 3.10 | 0.82 |
| Average | 0.15 | 0.52 | 2.18 | 2.66 |
| CPT of 10% Soybean Oil | | | 2.18 | |
| | | | 2min 11sec | |

**10% Spearmint Oil Emulsion**

| Trial | Control 1st Crossing | Control 2nd Crossing | Treatment 1st Crossing | Treatment 2nd Crossing |
|---|---|---|---|---|
| 1 | 0.15 | 0.52 | 2.98 | 4.97 |
| 2 | 0.30 | 0.70 | 29.67 | 44.13 |
| 3 | 0.68 | 1.00 | 87.05 | 87.48 |
| 4 | 0.92 | 0.08 | 1.77 | 23.25 |
| 5 | 0.33 | 1.00 | 56.60 | 75.50 |
| 6 | 0.50 | 0.65 | 55.80 | 55.83 |
| Average | 0.48 | 0.66 | 38.98 | 48.53 |
| CPT of 10% Spearmint Oil | | | 38.98 | |
| | | | 38min 59sec | |

**10% Thyme Oil Emulsion**

| Trial | Control 1st Crossing | Control 2nd Crossing | Treatment 1st Crossing | Treatment 2nd Crossing |
|---|---|---|---|---|
| 1 | 0.15 | 0.52 | 2.98 | 4.97 |
| 2 | 0.30 | 0.70 | 29.67 | 44.13 |
| 3 | 0.27 | 0.43 | 0.77 | 1.07 |
| 4 | 0.55 | 0.98 | 124.07 | 126.70 |
| 5 | 0.48 | 0.70 | 109.25 | 109.72 |
| Average | 0.35 | 0.67 | 39.37 | 57.32 |
| CPT of 10% Thyme Oil | | | 39.37 | |
| | | | 39min 22sec | |

**Supplemental Table S2** – Raw data in minutes collected for the tick on-arm assay

| ID | Calculated RI | Reference RI | RI DIFF | Spectrum similarity (%) |
|---|---|---|---|---|
| 4-Methyl-1,2,3,5,6-pentathiepane c | 1665 | 1646 | 19 | NA |
| Abieta-7,13-dien-3-one | 2311 | 2312 | 1 | 71 |
| Acoradiene<beta-> | 1469 | 1469 | 0 | 89 |
| Allyl hexanoate | 1074 | 1079 | 5 | 78 |
| Allyl methyl trisulfide | 1147 | 1138 | 9 | NA |
| Amorpha-4,11-diene | 1450 | 1449 | 1 | 71 |
| Aromadendrene | 1435 | 1439 | 4 | 94 |
| Atlantol<beta-> | 1602 | 1608 | 6 | 75 |
| Benzene acetaldehyde | 1030 | 1036 | 6 | 86 |
| Benzyl benzoate | 1781 | 1759 | 22 | 95 |
| Borneol | 1178 | 1165 | 13 | 94 |
| Bornyl acetate | 1305 | 1287 | 18 | 96 |
| Bourbonene<beta-> | 1398 | 1387 | 11 | 98 |
| Bulnesene<alpha-> | 1518 | 1509 | 9 | 72 |
| Cadina-1(6),4-diene<cis-> | 1454 | 1461 | 7 | 89 |
| Cadinene<alpha-> | 1536 | 1537 | 1 | 72 |
| Cadinene<delta-> | 1537 | 1522 | 15 | 71 |
| Cadinene<gamma-> | 1513 | 1513 | 0 | 84 |
| Calacorene<beta-> | 1558 | 1564 | 6 | 89 |
| Calamenene<cis-> | 1537 | 1528 | 9 | 96 |
| Camphene | 954 | 946 | 8 | 96 |
| Carvacrol | 1301 | 1298 | 3 | 90 |
| Carvone | 1255 | 1239 | 16 | 87 |
| Caryophyllene(E-) | 1421 | 1417 | 4 | 95 |
| Caryophyllene<14-hydroxy-4,5-dihydro-> | 1707 | 1706 | 1 | 74 |
| Caryophyllene<9-epi-(E)-> | 1466 | 1464 | 2 | 90 |
| Chavibetol acetate | 1537 | 1524 | 13 | 94 |
| Chrysanthenyl acetate<trans-> | 1247 | 1235 | 12 | 87 |
| Cineole<1,8-> | 1038 | 1026 | 12 | 93 |
| Cinnamaldehyde<E-> | 1282 | 1267 | 15 | 98 |
| Cinnamyl acetate<E-> | 1453 | 1443 | 10 | 96 |
| Citronellyl acetate | 1358 | 1350 | 8 | 93 |
| Citronellyl butanoate | 1533 | 1530 | 3 | 96 |
| Citronellyl formate | 1281 | 1271 | 10 | 86 |
| Citronellyl propanoate | 1449 | 1444 | 5 | 95 |
| Citronet | 1235 | 1234 | 1 | 85 |
| Cubebene<beta-> | 1392 | 1387 | 5 | 91 |
| Cubenol<1-epi-> | 1625 | 1627 | 2 | 82 |
| Cuparene | 1491 | 1504 | 13 | 89 |
| Curcumene<beta-> | 1516 | 1514 | 2 | 86 |
| Cyclooctasulfur | 2067 | 2041 | 26 | NA |
| Cymene<ortho-> | 1029 | 1022 | 7 | 97 |
| Dauca-4(11),8-diene | 1521 | 1530 | 10 | 84 |
| Diallyl disulfide | 1086 | 1080 | 6 | NA |
| Diallyl sulfide | 860 | 855 | 5 | NA |
| Diallyl tetrasulfide | 1556 | 1540 | 16 | NA |
| Diallyl trisulfide | 1312 | 1301 | 11 | NA |
| Dihydro carveol acetate<iso-> | 1338 | 1326 | 12 | 94 |
| Dihydro carvone<trans-> | 1205 | 1200 | 5 | 88 |
| Dimethyl trisulfide | 977 | 968 | 9 | NA |
| Eudesmol<7-epi-alpha-> | 1670 | 1662 | 8 | 86 |
| Eugenol | 1372 | 1356 | 16 | 96 |
| Funebrene<2-epi-alpha-> | 1384 | 1380 | 4 | 73 |
| Geranial | 1279 | 1264 | 15 | 79 |
| Germacrene B | 1562 | 1559 | 3 | 95 |
| Guaiol | 1600 | 1600 | 0 | 89 |
| Gurjunene<beta-> | 1429 | 1431 | 2 | 86 |
| Hepten-2-one<6-methyl-5-> | 988 | 981 | 7 | 86 |
| Hexenyl 3-methyl butanoate<3Z-> | 1235 | 1232 | 3 | 89 |
| Himachalol | 1652 | 1652 | 0 | 84 |
| Isocitral<E-> | 1186 | 1177 | 9 | 93 |
| Isoeugenol<E-> | 1460 | 1448 | 12 | 94 |
| Isopulegol<iso-> | 1153 | 1155 | 2 | 90 |
| Karahanaenone | 1154 | 1154 | 0 | 79 |
| Khusimone | 1597 | 1604 | 7 | 75 |
| Longifolene | 1403 | 1407 | 4 | 91 |
| Macrocarpene<(Z)-gamma-> | 1516 | 1512 | 4 | 73 |
| Mentha-2,8-diene<cis-meta-> | 983 | 983 | 0 | 90 |
| Menthol<iso-> | 1174 | 1179 | 5 | 89 |
| Menthol<neoiso-> | 1192 | 1184 | 8 | 88 |
| Menthone<iso-> | 1162 | 1158 | 4 | 88 |
| Menthyl acetate | 1301 | 1294 | 7 | 95 |
| Menthyl acetate<neo-> | 1280 | 1271 | 9 | 71 |
| Methyl (Z)-1-propenyl trisulfide | 1166 | 1159 | 7 | NA |
| Methyl communate<E-> | 2266 | 2257 | 9 | 74 |
| Mint sulfide | 1761 | 1740 | 21 | 91 |
| Muurola-4(14),5-diene<trans-> | 1492 | 1493 | 1 | 94 |
| Muurolene<alpha-> | 1494 | 1500 | 6 | 87 |
| Muurolol<alpha-> (=Torreyol) | 1648 | 1644 | 4 | 87 |
| Myrcene | 993 | 988 | 5 | 93 |
| Myrtanol acetate<trans-> | 1388 | 1385 | 3 | 86 |
| Neryl formate | 1280 | 1280 | 0 | 74 |
| Nootkatene | 1521 | 1517 | 4 | 78 |
| Ocimene<neo-allo-> | 1158 | 1140 | 18 | 77 |
| Octanol<3-> | 996 | 988 | 8 | 91 |
| Pentanone<4-hydroxy-4-methyl-2-> | 839 | 831 | 8 | 86 |
| Phellandrene<alpha-> | 1009 | 1002 | 7 | 98 |
| Phellandrene<beta-> | 1034 | 1025 | 9 | 96 |
| Phenyl ethyl propanoate<2-> | 1365 | 1351 | 14 | 86 |
| Phenyl ethyl tiglate<2-> | 1597 | 1584 | 13 | 93 |
| Pinene<alpha-> | 938 | 932 | 6 | 98 |
| Pulegone | 1246 | 1233 | 13 | 92 |
| Rose oxide<trans-> | 1115 | 1122 | 7 | 89 |
| Sabinene hydrate acetate<trans->(Ac vs. IPP) | 1262 | 1253 | 9 | 90 |
| Sabinene hydrate<trans->(IPP vs OH) | 1103 | 1098 | 5 | 87 |
| Safrole | 1299 | 1285 | 14 | 94 |
| Selinene<alpha-> | 1494 | 1498 | 4 | 95 |
| Terpinen-4-ol acetate | 1307 | 1299 | 8 | 70 |
| Terpinene<gamma-> | 1068 | 1054 | 14 | 98 |
| Terpineol<gamma-> | 1201 | 1199 | 2 | 92 |
| Terpinolene | 1103 | 1086 | 17 | 75 |
| Thuja-2,4(10)-diene | 938 | 953 | 15 | 71 |
| Unidentified e | 1382 | 1379 | 3 | NA |
| Valerianol | 1657 | 1656 | 1 | 87 |
| Zonarene | 1531 | 1528 | 3 | 91 |

**Supplemental Table S3.** Compound list with reported and expected retention index and spectrum similarity. NA Compound identification based on reported RI values Satyal et al. 2017
